# A Leaky False Pouch: Left Ventricle Pseudoaneurysm with Active Hemopericardium Detected on Cardiac Computed Tomography Angiography

**DOI:** 10.1177/11795468241249059

**Published:** 2024-04-27

**Authors:** Shiva Barforoshi, Chandana Shekar, Zoe Yu, Eugene Liu, Venkat Manubolu, Matthew J Budoff, Sion K Roy

**Affiliations:** 1 Department of Medicine, Harbor-UCLA Medical Center, Torrance, CA, USA; 2 Lundquist Institute, Harbor-UCLA Medical Center, Torrance, CA, USA

**Keywords:** Pseudoaneurysm, hemopericardium, left ventricle pseudoaneurysm, myocardial infarction, cardiac computed tomography angiography

## Abstract

Pseudoaneurysm is a rare but fatal complication of myocardial infarction (MI). With the advances in cardiovascular disease detection and treatments, fatal structural complications post-MI are now rare. When they occur, advanced diagnostic modalities can be used for early diagnosis, aiding surgical planning, and improving prognosis. In our case, post-MI left ventricle pseudoaneurysm complicated by hemopericardium was diagnosed using cardiac computed tomography angiography (CCTA). Use of attenuation measurement on CCTA helped diagnose active extravasation into the hemopericardium. This case highlights the high index of suspicion needed for rare but fatal complications post-MI and the utility of CCTA in their management.

## Introduction

Pseudoaneurysm is a rare but fatal complication of myocardial infarction (MI). Pseudoaneurysm is a type of incomplete myocardial rupture secondary to weakened myocardium and increased wall stress. It is seen in 23% of those experiencing fatal infarcts, and is twice as common after inferior infarction than anterior.^1^ Pseudoaneurysms are contained by either a portion of the scar, pericardium, or thrombus. Hemopericardium can result when blood leaks and collects between the ruptured myocardium and the pericardium.^2^ Fatal structural complications post-MI are now rare. However, when they occur, advanced diagnostic modalities can be used for early diagnosis, aiding surgical planning, and improving prognosis. We report the case of a patient with post-MI left ventricle pseudoaneurysm complicated by hemopericardium diagnosed using cardiac computed tomography angiography (CCTA).

## Case

A 42-year-old man with a past medical history of late anterior ST-elevation myocardial infarction (STEMI) who underwent cardiac catheterization 15 days prior with placement of a drug-eluting stent in the mid-left anterior descending artery presented with epigastric pain and progressive dyspnea. His prior hospital course was complicated by mild pericardial effusion, post-MI pericarditis, and left ventricular thrombus. He had been discharged on dual anti-platelet therapy and Rivaroxaban.

His cardiovascular exam was remarkable for pulsus paradoxus and jugular venous distension of 11 cm. Differential diagnosis was broad for post-MI complications including ventricular wall rupture, new mitral regurgitation, and pericardial tamponade. He underwent transthoracic echocardiography (TTE) which showed a moderate-sized circumferential pericardial effusion with cardiac tamponade, large non-mobile apical thrombus in the left ventricle, reduced left ventricle ejection fraction of 30%-35%, and no evidence of pseudoaneurysm despite use of Definity contrast imaging. Pericardiocentesis was subsequently performed which revealed a bloody effusion with 750 mL drained, and a pericardial drain was placed. Despite the lack of evidence on TTE, there was concern for a left ventricle pseudoaneurysm given the patient had a recent history of a late STEMI and was at high risk for structural complications. Therefore, cardiac computed tomography angiography (CCTA) was performed.

**Figure 1.**
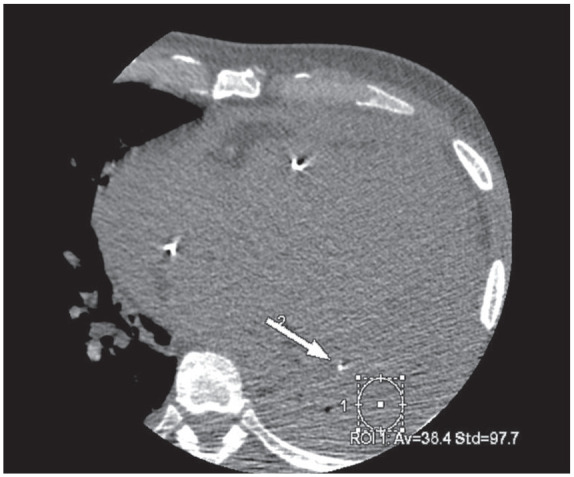
Axial view of coronary computed tomography angiography study without contrast. Arrow 2 indicates pericardial drain. Circle 1 shows the Hounsfield units in the surrounding area is 38.4, consistent with hemopericardium.

**Figure 2.**
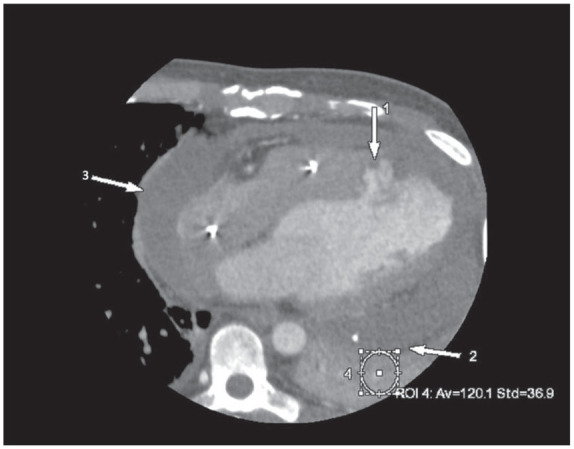
Axial view of coronary computed tomography angiography study with contrast. Arrow 1 indicates the pseudoaneurysm measuring 1.7 cm, which is contained by pericardium at arrow 2 noted at the neck of the apical septum of the left ventricle, consistent with hemopericardium. There is circumferential pericardial effusion at arrow 3. Circle 4 shows an increase in Hounsfield to 120, indicating active extravasation.

**Figure 3.**
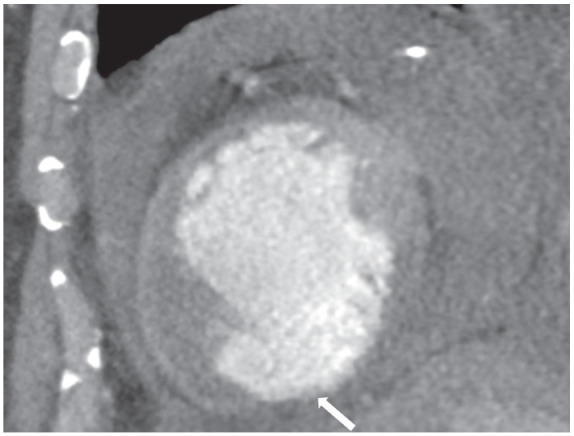
Short axis view of coronary computed tomography angiography study with contrast. Arrow indicates broad based aneurysm in the left ventricle.

On the non-contrast CCTA study, a large circumferential pericardial effusion was noted, most prominent on the lateral aspect of the left ventricle with 38.4 Hounsfield Units (HU) in the area which was consistent with hemopericardium (Figure 1). On the contrast CCTA study, the HU in the same area increased to 120, and a pseudoaneurysm, measuring 1.7 cm at the neck was noted at the apical septum of the left ventricle contained by the pericardium. The contrast-induced increase in HU in the hemopericardium indicated ongoing extravasation from the pseudoaneurysm into the hemopericardium (Figure 2). This is also noted on 3-Dimensional reconstruction of the left ventricular pseudoaneurysm (Figure 3). This was confirmed during surgery when the patient underwent repair of the left ventricular pseudoaneurysm with placement of a 0.8 cm × 9 cm xenoguard patch to prevent high risk of free wall rupture. Additionally, an extensive amount of friable and necrotic myocardium was resected on the left ventricular pseudoaneurysm. He underwent successful surgical repair of the aneurysm with no post-operative complications. He was discharged on dual anti-platelet therapy and Warfarin. Repeat TTE initially showed worsening ejection fraction of 10%-20% which later improved to 45%-50%, consistent with heart failure with improved ejection fraction, and he is on guideline-directed medical therapy with New York Heart Association class I symptoms. Given the persistence of thrombus on repeat imaging, he will continue on lifelong anticoagulation therapy. He continues to follow-up regularly with Cardiology on an outpatient basis.

## Discussion

It is crucial to differentiate pseudoaneurysms from true aneurysms, as the former have a higher risk of rupture leading to cardiac tamponade, shock, and death when compared to the benign course of the latter.^2^ While the wall of true aneurysms has all 3 layers of cardiac tissue and contain muscle tissue, pseudoaneurysm sacs are devoid of them which makes their rupture more likely. Pseudoaneurysms can also be differentiated from true aneurysms by their shape and location as well. True aneurysms have a broad-based neck, whereas the neck of the sac in pseudoaneurysms are narrow. Pseudoaneurysms occur most commonly following the occlusion of the left circumflex or right coronary artery, hence are located in the posterior or inferior walls. True aneurysms, however, are most often due to the occlusion of the left anterior descending artery and located in the apex or anterolateral wall.^3^

Rupture can happen in the very early phase post-MI, making the risk of sudden death due to a pseudoaneurysm rupture to be 30%-45%.^3^ Around 10% of cases may slowly develop over the years and be asymptomatic before rupturing. When symptoms do appear, they greatly vary in their presentation. In a report with 290 patients with left ventricle pseudoaneurysm, 36% of patients presented with symptoms of congestive heart failure, 30% with chest pain, and 25% with dyspnea. Sudden death was the presenting sign in 3% of cases. More than two-thirds of patients had a murmur. Pleuritic chest pain, cough, and signs of an ongoing inflammatory process can also lead to a wrong diagnosis of a pulmonary process.^4^ Few non-specific signs and symptoms have been reported in other case reports, thus calling for a high degree of suspicion. Physical examination, ECG, and chest X-ray might be inconclusive as well. Cardiomegaly, pleural effusion, and a discrete bulge on the cardiac border on the chest X-ray have been described. Persistent ST-segment elevation, Q waves, or nonspecific ST abnormality may be present on the ECG.^5^

Given the wide array of symptomatology, diagnosis can be challenging. Cardiac imaging modalities are therefore often crucial in aiding diagnosis and advances in noninvasive imaging have improved the ability to distinguish pseudoaneurysm from aneurysm. Imaging characteristics that distinguish pseudoaneurysm from true aneurysm include a narrow neck when compared to the fundus, inferior-posterior location, saccular contour of the aneurysm, and sharp myocardial discontinuity of the endocardial border at the base of the pseudoaneurysm. While echocardiography can identify the abnormality in 90% of cases, given its measurements are 2 dimensional, it can only give a definite diagnosis in 25%-33% of cases. Both CCTA and cardiac MRI allow for visualization of any heart plane, thereby showing segments that are challenging to see on echocardiography. Cardiac MRI has high tissue characterization and spatial resolution helping distinguish pseudoaneurysm from true aneurysm.^3^ MRI can also exclude pseudoaneurysm by detecting presence of epicardial fat adjacent to an aneurysmal cavity and delayed enhancement seen in infarcted true aneurysmal myocardium.^2^

This case highlights the utility of CCTA, a relatively non-invasive and widely-available imaging modality, for identification of pseudoaneurysm as well as active leak, thus aiding in earlier diagnosis and surgical planning. CCTA was able to detect the patient’s pseudoaneurysm that TTE with Definity could not. CCTA allows for accurate delineation of the defect and differentiation from a true aneurysm, given its good spatial resolution.^5^ It also provides excellent visualization of the coronary arteries, LV myocardium, and bypass grafts. CCTA findings that help to distinguish a false aneurysm includes lack of surrounding coronary arteries and a narrow orifice that leads into a saccular aneurysm.^3^ An ECG-gated CT scan can provide information about the presence of pseudoaneurysm, its size, location, origin, and myocardial enhancement pattern. It can also diagnose calcification in the wall of chronic pseudoaneurysms. CCTA can also aid in the diagnosis of impending rupture as well. Higher attenuation of the hemopericardium on a CCTA when compared to the non-contrast CT scan helps diagnose an actively leaking aneurysm due to the mixing of contrast with blood.^6^ In conclusion, CCTA allows for earlier detection and better understanding of pseudoaneurysm characteristics, thus aiding surgical planning and giving a chance to improve prognosis.

## Learning Objectives

1. To have a high index of suspicion for rare but fatal complications post-MI, including pseudoaneurysm.
2. To value the clinical utility of cardiac computed tomography angiography as first line non-invasive imaging modality for cardiac pseudoaneurysm given its diagnostic accuracy and guidance of treatment strategy, including higher attenuation of hemopericardium to detect actively leaking aneurysm.
